# Thermal Conductivity above 2,000 W/m·K in Boron Arsenide by Nanosecond Transducer-Less Time-Domain Thermoreflectance

**DOI:** 10.34133/research.0971

**Published:** 2025-10-27

**Authors:** Hong Zhong, Ying Peng, Feng Lin, Ange Benise Niyikiza, Fengjiao Pan, Chengzhen Qin, Jinghong Chen, Viktor G. Hadjiev, Liangzi Deng, Zhifeng Ren, Jiming Bao

**Affiliations:** ^1^ Department of Electrical & Computer Engineering and Texas Center for Superconductivity at the University of Houston (TcSUH), University of Houston, Houston, TX 77204, USA.; ^2^ Department of Physics and Texas Center for Superconductivity at the University of Houston (TcSUH), University of Houston, Houston, TX 77204, USA.; ^3^ Materials Science and Engineering, University of Houston, Houston, TX 77204, USA.; ^4^ Department of Mechanical Engineering and Texas Center for Superconductivity at the University of Houston (TcSUH), University of Houston, Houston, TX 77204, USA.

## Abstract

Time-domain thermoreflectance (TDTR) has been a standard technique for measuring thermal conductivity (κ) for more than 3 decades, yet its reliance on femtosecond lasers and metal transducers has limited its broader adoption in the materials community. Recent attempts to eliminate the metal layer have achieved partial success but have been hampered by dominant reflectance from photoexcited carriers, arising from the continued use of femtosecond pump and 800-nm probe pulses. Here, we introduce a nanosecond transducer-less TDTR (tl-TDTR) method that overcomes this challenge. Using ~80-ns pump pulses and a 450-nm continuous-wave probe, we suppress carrier-induced negative transients, yielding positive signals characteristic of pure thermoreflectance. Thermal conductivity is extracted via heat transport simulations and direct time-domain curve fitting. The method is validated on benchmark semiconductors (Si, Ge, InP) and cross-checked on Si and diamond using an Al-film transducer. Applied to cubic boron arsenide crystals, the technique reveals room-temperature κ exceeding 2,000 W/m·K—comparable to single-crystal diamond—and confirmed by traditional TDTR on the same samples. Raman, photoluminescence (PL), and PL lifetime measurements indicate high crystal quality. Sub-10-ns lifetimes remain shorter than expected for an indirect bandgap semiconductor, suggesting headroom for further κ improvement. The observed ~1/*T*^2^ temperature dependence indicates dominant 4-phonon scattering. Nanosecond tl-TDTR thus provides a rapid, nondestructive route to assess semiconductor thermal conductivity.

## Introduction

Traditional time-domain thermoreflectance (TDTR) uses femtosecond pump–probe pulses and a thin metal film transducer to convert absorbed pump energy into heat, with the delayed probe sensing the transducer’s temperature-dependent reflectivity [1–4]. While TDTR has been a well-established technique for over 30 years, its adoption remains largely confined to a few specialized laboratories due to the high cost of femtosecond lasers and the complexity of data analysis, as reflected in recent cubic boron arsenide (c-BAs) thermal conductivity reports [5–7]. The metal transducer also introduces an additional and time-consuming step, requiring careful preparation to ensure a highly conductive thermal interface with the sample. This layer adds uncertainty due to unknown interfacial thermal conductance and can hinder subsequent material characterization or device testing, while chemical removal risks damaging the sample. Moreover, conventional femtosecond TDTR is typically operated in the frequency domain with high-frequency modulation and lock-in detection, making measurements slow and real-time thermal conductivity extraction challenging [2,3].

As such, there has been strong interest in developing a transducer-less version of traditional TDTR (tl-TDTR) by simply eliminating the metal transducer while keeping the same femtosecond lasers and detection system [8–10]. However, these efforts have faced important theoretical and practical challenges and have met with limited success [8–10]. A major obstacle, recognized early on, is that the pump-induced transient reflectance of the probe pulse in bare samples is influenced not only by temperature changes but also by photo-excited charge carriers [8–10]. In metal transducers such as Al or Au, these carriers have ultrashort lifetimes—on the order of femtoseconds—allowing rapid relaxation into heat. As a result, changes in probe reflectivity are directly related to temperature variations in the metal transducer, i.e., thermoreflectance. In contrast, charge carriers in semiconductors have longer lifetimes. They are excited almost instantaneously by the pump and persist well after its passage, causing ultrafast transient reflectance to be dominated by electronic rather than purely thermal effects [8–10]. This explains why femtosecond time-resolved reflectance has historically been used to study coherent electronic states and carrier dynamics [11–14]. To suppress electronic contributions in femtosecond tl-TDTR and recover a dominant thermoreflectance signal, researchers have tried long pump–probe delays (>ns) or low modulation frequencies [8–10]. Neither approach fully removes the electronic component. While long delays have been somewhat effective in measuring the thermal conductivity of Si and Ge [8–10], the signal weakens sharply at such timescales and often decays to undetectable levels after femtosecond excitation, undermining the advantages of ultrafast time resolution [10].

In this work, we demonstrate a nanosecond tl-TDTR technique that overcomes these limitations. Our strategy is to use longer, lower-intensity pump pulses (very low free carrier plasma edge) and a short-wavelength probe of states above the hot carrier energy and away from the bandgap edge and critical points [15]. This approach allows us to circumvent the carrier-induced reflectance contribution, producing fully positive temperature-driven traces. Accuracy is established via κ extraction on reference materials and self-consistent measurements with and without an Al transducer. We then rapidly map κ in c-BAs, identifying regions exceeding 2,000 W/m·K and confirming these values with femtosecond TDTR on the same crystals. Together, these results show that nanosecond tl-TDTR provides a fast, versatile, and minimally invasive approach to quantify heat transport across a wide range of semiconductors.

## Results and Discussion

**Fig. 1.**
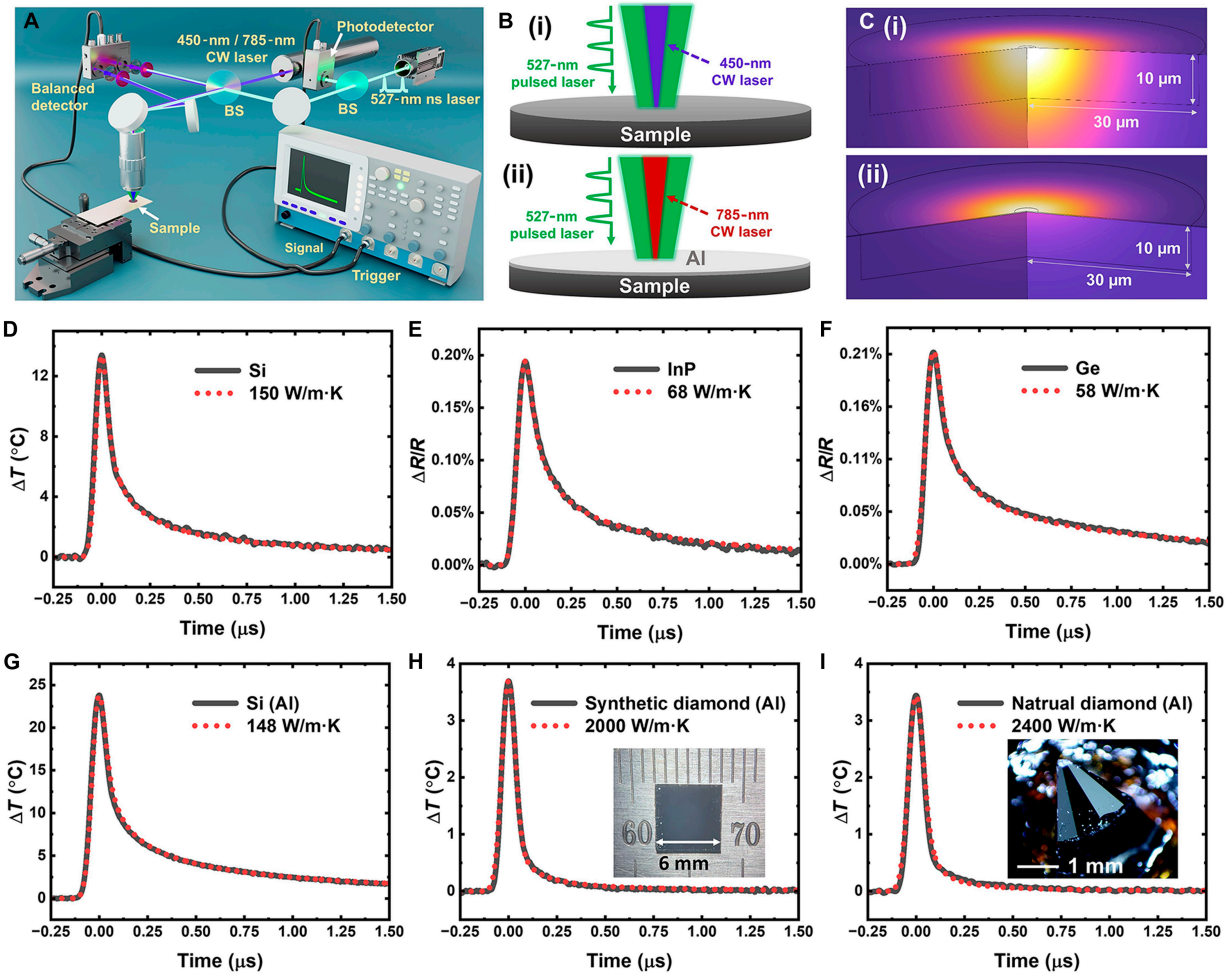
Demonstration of nanosecond TDTR with well-characterized materials, both without and with an Al film as a transducer. (A and B-i) Schematics of the experimental setup for nanosecond tl-TDTR. (C-i) COMSOL simulation model (actual simulation domain: 300 μm tall and 500 μm in radius). (D to F) Representative nanosecond tl-TDTR traces of Si, InP, and Ge (black solid lines) and corresponding COMSOL fits (red dotted lines). (A, B-ii, C-ii) Schematics and COMSOL simulation model for nanosecond TDTR with an Al transducer. (G to I) Representative nanosecond TDTR traces with Al coating for Si, synthetic diamond, natural single crystal diamond (black solid lines), and their COMSOL fits (red dotted lines). Insets in (H) and (I) are optical pictures of synthetic and natural diamonds coated with Al films.

Figure 1A and B-i illustrates the experimental setup for tl-TDTR. A 527-nm nanosecond laser (~80 ns, 1 kHz repetition rate) serves as the pump, while a 450-nm continuous-wave (CW) laser functions as the probe. Both beams are focused on the same spot through a microscope objective, with pump and probe spot sizes of ~30 μm and ~5 μm, respectively. The pump power is varied from 0.1 to 2.5 mW depending on the material’s reflectivity and absorbance, while the probe power is maintained below 2 mW. To achieve high time resolution and signal gain, we use a fast balanced photodetector to continuously monitor the reflected probe, while a digital oscilloscope (Agilent/Keysight MSO9404A) rapidly acquires and averages the signal over hundreds of iterations. The high repetition rate of the pump allows for efficient averaging over thousands of cycles, ensuring a strong signal-to-noise ratio without excessive measurement time. By eliminating the mechanical pump–probe delay rail, high-frequency pump modulation, and lock-in detection of the reflected probe, tl-TDTR enables real-time acquisition of thermoreflectance traces. Multi-physics COMSOL modeling is employed to simulate optical heating and subsequent heat transfer dynamics (Fig. 1C-i). It is flexible to accommodate various configurations, including different pump spot sizes and the presence or absence of a metal film transducer. Key modeling parameters, such as the optical absorption coefficient and thermoreflectance coefficient, are obtained from literature or measured in the laboratory [16–24]. The pump beam is modeled as a Gaussian beam with diameter and power values derived from experimental measurements (see Methods and the Supplementary Materials for details).

Figure 1D to F shows representative nanosecond tl-TDTR traces for Si, InP, and Ge. All 3 exhibit entirely positive reflectance signals throughout the measurement window, in sharp contrast to previous femtosecond tl-TDTR studies, which were dominated by negative signals due to photoexcited charge carriers [8–10]. For Si, the relative change in reflectance (Δ*R*/*R*) has been converted to a temperature change Δ*T* = Δ*R*/(*R*·C_TR_) using its thermoreflectance coefficient *C*_TR_ = 1.3 × 10^−4^ K^−1^ (Fig. S7). Each trace exhibits a sharp reflectance peak immediately after pump pulse excitation, followed by a gradual decrease, indicating rapid heating by the pump pulse and subsequent cooling. The width of the initial peak is largely determined by the pump pulse duration, while the subsequent cooling rate reflects the material’s thermal conductivity. The experimental curves are well-fitted using COMSOL simulations (dotted lines), yielding κ values of 150, 68, and 58 W/m·K for Si, InP, and Ge, respectively—fairly consistent with reported values [9,10,25]. It is important to note that the actual value of the thermoreflectance coefficient will not affect the heat transport dynamics; rather, it influences the sensitivity of temperature measurement, similar to the role of the metal film in femtosecond TDTR [2]. Therefore, we normalize the Δ*R*/*R* traces during COMSOL fitting and are able to extract the thermal conductivities of Ge and InP without knowing their thermoreflectance coefficients.

Nanosecond tl-TDTR is also compatible with thin metal transducers, which are required when a semiconductor’s bandgap exceeds the pump laser energy. To demonstrate this flexibility and further confirm the accuracy of nanosecond tl-TDTR, we chose Si, synthetic diamond, and natural single-crystal diamond for validation. We deposited a thin Al layer on them and selected a 785-nm probe laser because Al exhibits a larger thermoreflectance temperature coefficient at that wavelength (Fig. 1A and B-ii) [2]. This thin Al film can be modeled in COMSOL by introducing an interfacial thermal conductance as a fitting parameter (Fig. 1C-ii) [2]. Figure 1G to I shows nanosecond TDTR traces for Si and diamond along with COMSOL fits for all 3 samples. We obtained the same κ for silicon and high values of 2,000 and 2,400 W/m·K for synthetic and natural diamonds, respectively, in agreement with accepted values [26]. We refer to this variation as “nanosecond TDTR”, in contrast to the conventional femtosecond TDTR. These results further validate the accuracy of nanosecond tl-TDTR across a wide range of thermal conductivities and demonstrate its versatility for characterizing both thin films and wide-bandgap semiconductors.

The all-positive reflectance observed in our nanosecond tl-TDTR arises from a careful selection of pump and probe parameters that effectively overcome the negative reflectance typically induced by photoexcited free carriers in femtosecond tl-TDTR [8–10]. First, the ~100-ns pump pulse used in our setup has a much lower peak intensity than femtosecond pulses. Combined with the significantly faster carrier diffusion relative to thermal diffusion, this greatly reduces the carrier concentration during excitation. For example, in silicon—a benchmark material for tl-TDTR—the electron diffusion coefficient is 35 cm^2^/s, roughly 40 times higher than its thermal diffusivity. Over the course of a 100-ns pulse, the carrier diffusion length reaches ~19 μm, well beyond the optical penetration depth of 1.2 μm. Moreover, our average pump power is 0.2 mW at 1 kHz, compared to ~20 mW at 80 MHz in femtosecond systems [2], leading to a peak intensity nearly 1,000 times lower. Carrier concentrations are further reduced through both radiative and nonradiative recombination [9]. Second, we employ a shorter-wavelength probe at 450 nm instead of the conventional ~800 nm, which strongly suppresses carrier-induced reflectivity. Free-carrier reflectance is negative and scales with the square of the wavelength (λ^2^) [9,10,27], but in practice diminishes even more rapidly at shorter wavelengths. In silicon, free-carrier contributions to reflectance become negligible at 450 nm or below [12,27]. Meanwhile, the nanosecond pulse duration still enables sufficient heat accumulation without significant dissipation, and the thermoreflectance coefficient increases at shorter probe wavelengths [15,28]. As a result, the negative transient reflectance commonly observed in femtosecond tl-TDTR is replaced by a purely positive thermoreflectance signal in our nanosecond tl-TDTR.

Due to the all-positive transient reflectance signals—similar to those observed with metal transducers in traditional TDTR and in nanosecond TDTR (Fig. 1G to I)—we directly applied heat transport simulations to fit the tl-TDTR data and extract thermal conductivity. This approach aligns with the strategy previously employed in tl-TDTR [9,10]. By focusing on the dominant positive signal at long pump–probe delays or low modulation frequencies, low-uncertainty thermal conductivity values can be obtained in materials like silicon [9,10]. In fact, in some extreme cases, thermal conductivity of silicon has even been extracted from purely negative transient reflectance signals, albeit with higher uncertainty [9]. A closely related methodology is found in transducer-less transient thermal grating techniques, where thermal decay is directly fitted using heat transport models just 1 ns after the dominant electrical response, yielding high thermal conductivity values for c-BAs [11]. These precedents support the validity of our approach, demonstrating that it is both accurate and justified to extract thermal conductivity from the fully positive transient reflectance signals in nanosecond tl-TDTR, particularly for high-κ materials like c-BAs.

**Fig. 2.**
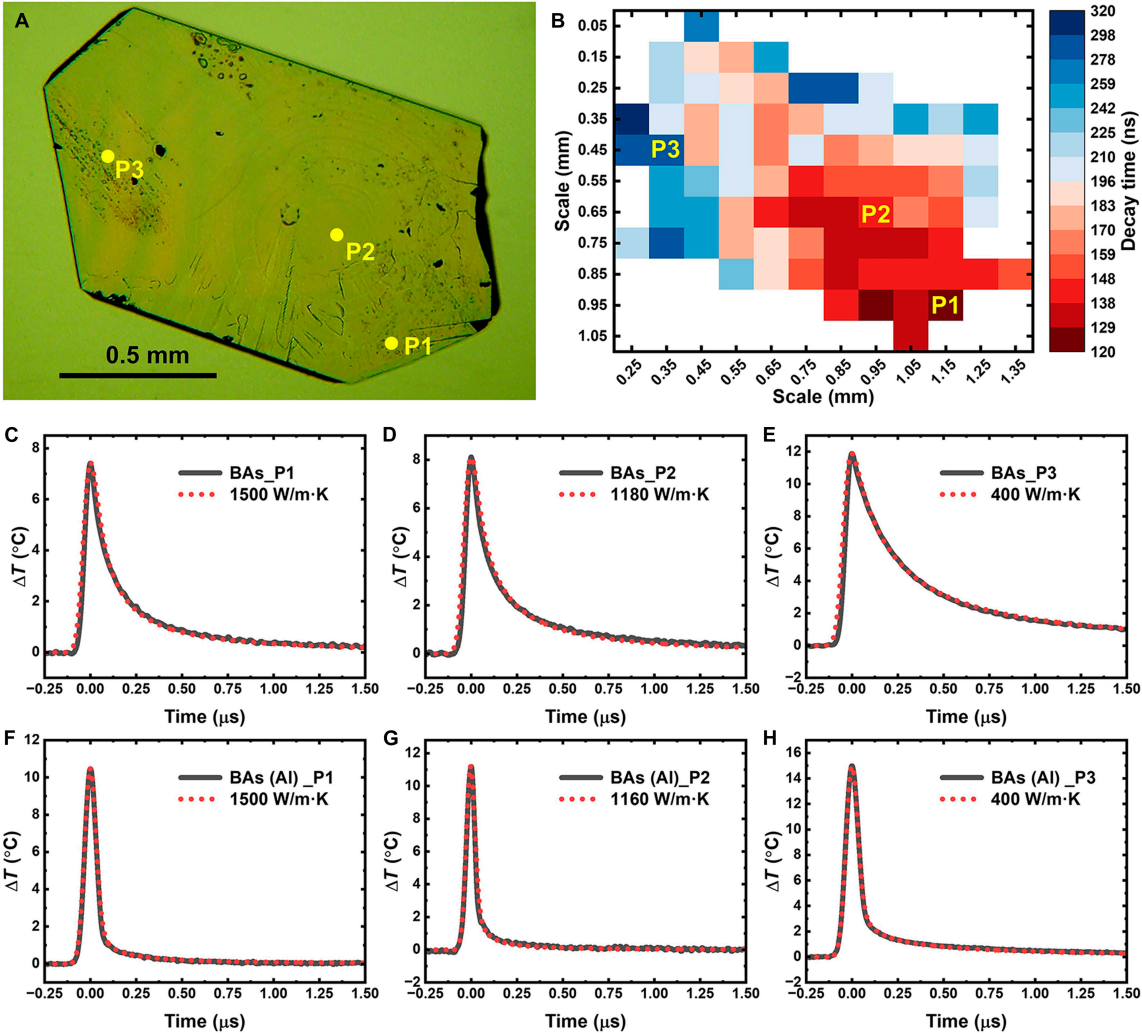
Nanosecond TDTR measurements of c-BAs with and without an Al transducer. (A) Optical image. The thickness of the crystal is ~195 μm. (B) tl-TDTR decay time map. (C to E) tl-TDTR traces (black solid lines) and corresponding COMSOL fits (red dotted lines) for 3 spots with thermal conductivities of 1,500 (C), ~1,180 (D), and 400 W/m·K (E). (F to H) Nanosecond TDTR traces (black solid lines) and COMSOL fits (red dotted lines) with Al transducer for the same 3 spots.

One key advantage of nanosecond tl-TDTR is its ability to rapidly assess relative thermal conductivity in real time by comparing cooling rates—faster cooling indicates higher conductivity. Given the inherent nonuniformity in c-BAs samples, we first performed a spatial scan of cooling rates using tl-TDTR and then selected specific regions for detailed COMSOL fitting. Figure 2A shows a scanning electron microscopy (SEM) image of a new c-BAs crystal, while Fig. 2B presents a manually acquired cooling rate map, where the cooling rate is defined as the time taken for the reflectance signal to decay from its peak to half its value. Prior to measurement, the surface was treated with acid and polishing cloth to remove surface oxides and debris, and flat, smooth regions were chosen for tl-TDTR analysis. Three representative points (P1, P2, and P3) were selected for further study. Figure 2C to E shows the corresponding tl-TDTR traces and COMSOL fits, revealing that spot P1 exhibits a thermal conductivity of 1,500 W/m·K—exceeding the theoretical prediction of 1,300 W/m·K. The temperature change Δ*T* was calculated using a measured thermoreflectance coefficient of approximately 3 × 10^−4^ K^−1^ at 450 nm, which remains nearly constant over the 300 to 400 K range (Fig. S8). To validate this high thermal conductivity, we deposited a 100-nm Al layer, switched the probe to a 785-nm laser, and remeasured the same locations using nanosecond TDTR. Figure 2F to H displays the resulting traces and COMSOL fits, confirming that all 3 spots yielded consistent thermal conductivity values. Given the small temperature rise during measurement, both the thermal conductivity and all input parameters were treated as constants.

**Fig. 3.**
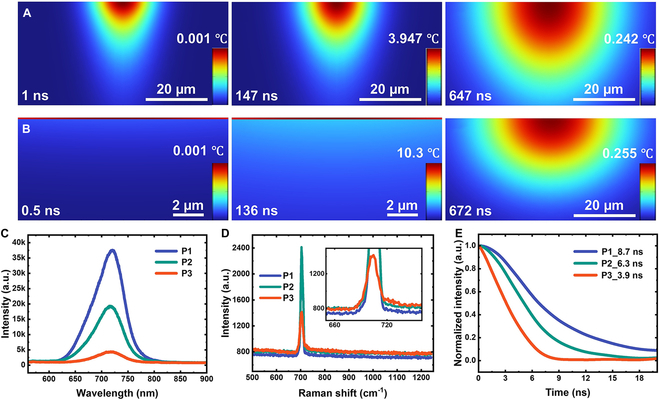
Comparison of nanosecond tl-TDTR and TDTR simulations without and with an Al transducer, and optical characterization of c-BAs. (A and B) COMSOL simulations for spot P1 of c-BAs without (A) and with (B) an Al transducer. The diameter of pump beam is 24 μm, and its pulse width is 70 ns. (C to E) Photoluminescence (PL), Raman, and PL lifetime measurements for spots P1, P2, and P3.

Figure 2 also reveals that nanosecond tl-TDTR and TDTR traces exhibit markedly different heat transport dynamics. With the Al transducer, the TDTR peak closely follows the pump pulse, producing a sharp response. The temperature drops rapidly after the peak, whereas in tl-TDTR, the temperature decreases much more gradually. This difference arises from the distinct heating mechanisms by the pump laser pulse. Nanosecond tl-TDTR involves volumetric heating [8], as the pump pulse penetrates to a depth of ~20 μm, based on ultraviolet–visible (UV–Vis) absorbance data [23]. In contrast, nanosecond TDTR with a metal transducer involves surface heating due to the extremely shallow optical penetration depth. In fact, in traditional femtosecond TDTR modeling, the metal transducer is often treated as a mathematically infinitesimal layer. As a result, heat rapidly accumulates at the metal film and dissipates through interfacial thermal conductance. This contrast is clearly illustrated in the COMSOL simulations in Fig. 3A and B, where the temperature of the Al film is uniform and significantly higher than that of the c-BAs crystal during the heating by pump pulse. In both cases, the rate of temperature decay reflects the underlying thermal conductivity of the materials. However, because the cooling of the Al transducer is also influenced by interfacial thermal conductivity, tl-TDTR provides a more direct measurement of the BAs crystal’s thermal conductivity.

The significantly higher thermal conductivity of P1 compared to P2 and P3 suggests that P1 has a lower level of impurities or defects. To verify this, we measured and compared their photoluminescence (PL) and Raman spectra (performed before Al evaporation), both of which are commonly used to identify regions with high thermal conductivity. Figure 3C presents the PL spectra of the 3 spots. P1 exhibits the strongest PL intensity, followed by P2, while P3 has the weakest. All 3 spectra are dominated by the bandgap PL peak centered at 720 nm, indicating the overall high quality of the sample [23,29]. Figure 3D shows the Raman spectra from 200 to 1,200 cm^−1^. P1 displays the lowest Raman background and the weakest Fano lineshape of the longitudinal optical (LO) phonon at 700 cm^−1^, further supporting its superior crystal quality [6,11]. Based on the PL and Raman data, it is evident that P1 has the fewest impurities or defects. This higher crystal quality is what enables P1 to achieve the highest thermal conductivity. To obtain a better understanding of the crystal quality of P1, we measured the PL lifetime—an established parameter for assessing silicon quality but not yet reported for c-BAs [30]. A longer PL lifetime indicates higher crystal quality, characterized by fewer defects or impurities and reduced nonradiative recombination. For PL lifetime measurements in c-BAs, we used a nanosecond laser (3 to 5 ns, 532 nm) to excite the sample and a fast photomultiplier tube (PMT) to detect PL signals [31]. The time-resolved PL traces of the 3 spots are shown in Fig. 3E: P1 exhibits the longest PL lifetime, while P3 has the shortest. Overall, these nanosecond-scale lifetimes are comparable to those of direct bandgap semiconductors but are significantly shorter than those of high-quality indirect bandgap semiconductors such as Si and GaP [30,32]. Given its longer PL lifetime, we conclude that P1 is expected to exhibit higher thermal conductivity than P2 and P3.

**Fig. 4.**
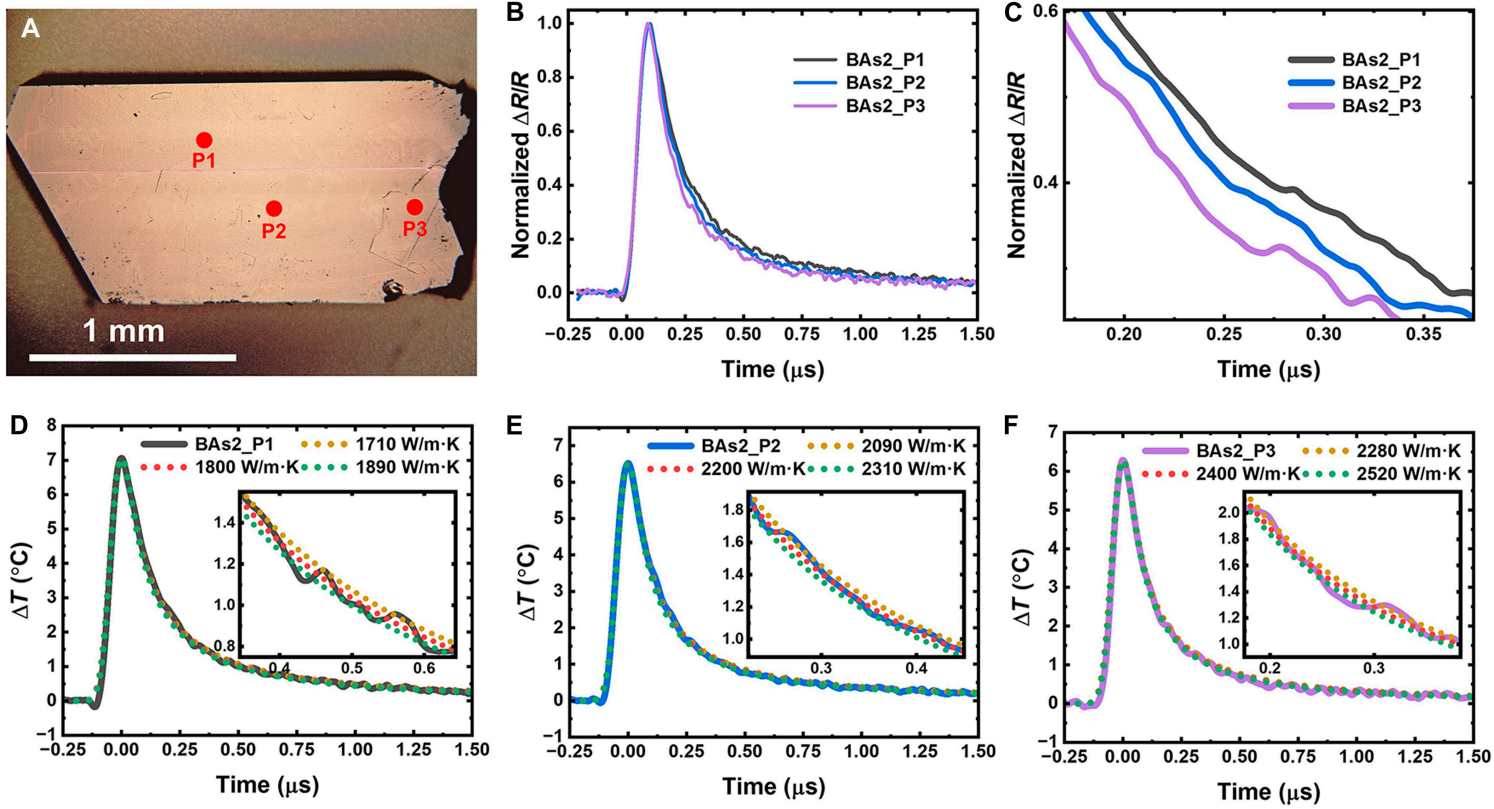
c-BAs crystal with thermal conductivity >2,000 W/m·K. (A) Image of a high-quality c-BAs showing 3 high-κ spots. The thickness of the crystal is ~215 μm. (B and C) Normalized tl-TDTR traces for the 3 spots, illustrating different cooling rates. (D to F) COMSOL fits (dotted lines) for the 3 tl-TDTR traces. Fits with κ ±5% are also included for comparison.

To further validate this ultrahigh thermal conductivity, we surveyed several additional crystals using the rapid tl-TDTR technique. Multiple spots with thermal conductivity exceeding 1,500 W/m·K were quickly identified, with some surpassing this value even further. Figure 4A shows an optical image of a high-quality sample, and Fig. 4B and C displays normalized tl-TDTR traces from 3 spots exhibiting high thermal conductivity. They exhibit very fast but different temperature cooling speeds. Figure 4D to F shows their corresponding COMSOL fits, revealing even higher thermal conductivities of 1,800, 2,200, and 2,400 W/m·K. To illustrate the uncertainty in thermal conductivity measurements, we also include COMSOL fits with values adjusted by ±5%. As shown, deviations of 5% result in imperfect fits, highlighting the sensitivity and accuracy of our measurements.

As a newly developed technique, it is essential to evaluate the uncertainty and accuracy of nanosecond tl-TDTR. Following standard TDTR methodology, we first assess the sensitivity of the thermal response to key experimental parameters and then quantify their impact on the extracted thermal conductivity [2,8–10]. The most critical parameter across all TDTR approaches is the pump spot size [2,8–10]. In our nanosecond tl-TDTR setup, additional relevant parameters include the pump pulse width and the optical absorption coefficient. Figure S9 shows the sensitivity of nanosecond tl-TDTR signal (Δ*R*/*R* or Δ*T*) to these 3 parameters. As expected, the signal is most sensitive to the pump spot size, while sensitivities to absorption coefficient and pulse width are significant only during the pump excitation period and negligible afterward. Figure S10 illustrates the corresponding uncertainty in the extracted thermal conductivity of silicon: A 2% uncertainty in pulse width results in a 1% error in conductivity. The absorption coefficient has the weakest effect: Even with a 22% uncertainty, the resulting error in conductivity remains below 2%. Similar uncertainty analysis for c-BAs, shown in Fig. S11, yields results comparable to those of silicon. In practice, we use a 3-mm silicon infrared window (Thorlabs WG80530) as a reference to further calibrate the pump spot size since we have verified its thermal conductivity of 147 W/m·K via the laser flash method. Given the minimal impact of absorption coefficient and the combined uncertainties from pump spot size, pulse width, and fitting, we estimate the total uncertainty in the extracted thermal conductivity of c-BAs to be approximately 10%.

The observation of thermal conductivity exceeding 2,000 W/m·K is unexpected but can still be understood from an experimental perspective. First, the seemingly excellent agreement between experiment and theory is not always consistent—exceptions exist. For example, a recent study measured the thermal conductivity of c-BAs with a silicon impurity concentration of 3 × 10^19^ cm^−3^ to be 920 W/m·K [33]. However, theoretical predictions suggest that it should be less than half of this value [11,34]. This discrepancy indicates that theoretical models can significantly underestimate thermal conductivity, suggesting that in ultrapure c-BAs, the actual thermal conductivity could far exceed the theoretical limit of 1,300 W/m·K. Second, the samples in this study were synthesized using an ultrapure arsenic source with significantly reduced impurity levels. Our previous secondary ion mass spectrometry analysis identified Si, C, and O as the primary impurities—mainly originating from the arsenic source [33]. Since then, we have systematically purified the As precursor, which has led to our recent observations of ultrahigh thermal conductivities of 1,500 and >2,000 W/m·K [35,36]. Finally, based on PL lifetime measurements, even spot P1 cannot yet be classified as ultrapure since the measured lifetime remains below hundreds of nanoseconds, which is expected for high-purity indirect bandgap semiconductors [30,32].

The high thermal conductivity values measured using our nanosecond tl-TDTR either are consistent or have been independently confirmed with traditional femtosecond TDTR methods by multiple groups. A recent paper by Wilson’s group at UC Riverside using crystals from Ren’s group reported a thermal conductivity of approximately 1,500 W/m·K in c-BAs [35]. The c-BAs crystal shown in Fig. 2 was actually grown using the same purified arsenic source as the samples used in their study, synthesized in Dr. Ren’s laboratory. Their work also identified 4-phonon scattering as the dominant scattering mechanisms, based on temperature-dependent thermal conductivity measurements. We performed the same measurements on spot P1 using nanosecond tl-TDTR, and Fig. S12 compares our results with those from UC Riverside, showing excellent agreement in both absolute conductivity and its nearly 1/*T*^2^ temperature dependence. Furthermore, thermal conductivity values exceeding 2,000 W/m·K were recently reported in a preprint by Liao’s group [36], in collaboration with Ren’s group. Using femtosecond TDTR, they not only validated our results on the same crystal shown in Fig. 4 but also independently confirmed the high thermal conductivity in the synthetic diamond sample presented in Fig. 1H (Fig. S13). These consistent findings and independent validations using traditional TDTR strongly support the reliability of the nanosecond tl-TDTR technique.

## Conclusion

In conclusion, we developed a nanosecond tl-TDTR that rapidly screens and quantifies semiconductor thermal conductivity without a metal transducer. By combining a nanosecond pump with a short-wavelength probe, we suppress carrier-induced negative reflectance and enhance thermoreflectance, producing all-positive traces suitable for direct heat transport fitting. Depending on bandgap and thermoreflectance, other pump–probe wavelengths may be optimal; UV nanosecond excitation is needed for wide-bandgap materials. The same setup readily converts to conventional nanosecond TDTR with a metal transducer when a UV pump is unavailable. Using this method, we measured κ in c-BAs exceeding 2,000 W/m·K, attributable to reduced impurities from purified arsenic sources and supported by increased PL lifetimes. The ~1/*T*^2^ dependence confirms dominant 4-phonon scattering. Given frequent correlations between lattice thermal conductivity and carrier mobility, these results also suggest potential for ultrahigh mobility in c-BAs. Determining the ultimate limits of κ and mobility will require further work. Beyond advancing fundamental understanding, nanosecond tl-TDTR enables high-throughput, nondestructive thermal screening for next-generation electronic and thermal management materials.

## Methods

### COMSOL multiphysics simulation of heat transfer for thermal conductivity calculation

Commercial software COMSOL Multiphysics was used to simulate the laser heating process and extract the thermal conductivity of BAs, Si, InP, Ge, and diamond. The Heat Transfer module was employed to model the interaction between the incident laser pulse and the materials. The laser source was defined with Gaussian spatial and temporal distributions. The input parameters included the specific heat capacity, absorption coefficient, density, and thermal conductivity of each material, as summarized in Table S1. For aluminum-coated samples, a Thermal Contact module was added to account for interfacial heat transfer.

The heat source in the simulation was a 527-nm nanosecond pulsed laser. A portion of the incident power was reflected at the sample surface, while the remainder was absorbed according to Beer–Lambert’s law. The laser beam was modeled as a Gaussian pulse in both space and time, as described by Eqs. (1) and (2), which define the temporal and spatial distributions, respectively.

$$f\left(t\right)=\frac{A}{\frac{{\text{σ}}_{t}}{2\sqrt{\text{ln}2}}\sqrt{2\pi }}\text{exp}\;\left(-\frac{t^{2}}{2{\left(\frac{{\text{σ}}_{t}}{2\sqrt{2\text{ln}2}}\right)}^{2}}\right)$$ (1)

$$g\left(x,y\right)=\frac{B}{{\left(\frac{{\text{σ}}_{xy}}{2\sqrt{\text{ln}2}}\right)}^{2}2\pi }\text{exp}\;\left(-\frac{x^{2}+y^{2}}{2{\left(\frac{{\text{σ}}_{xy}}{2\sqrt{2\text{ln}2}}\right)}^{2}}\right)$$ (2)

Since the geometry was built in polar coordinates, *x*^2^ + *y*^2^ was replaced by *r*^2^. Integration over these equations yielded the total laser energy.

$$∭f\left(t\right)g\left(x,\;y\right)\text{dtdxdy}=\text{Absorbed energy from each pulse}$$ (3)

By accounting for surface reflection, the instantaneous laser power density reaching the sample surface was expressed as Eq. (4):

$$\begin{array}{c} \;\frac{\frac{p}{f}\alpha \times {\left(2\sqrt{2\text{ln}2}\right)}^{3}}{\sigma {{}_{xy}}^{2}{\text{σ}}_{t}{\left(2\pi \right)}^{1.5}}\;\text{exp}\left(-\frac{{\left(t-t_{0}\right)}^{2}}{2{\left(\frac{{\text{σ}}_{t}}{2\sqrt{2\text{ln}2}}\right)}^{2}}\right)\\ \;\text{exp}\left(-\frac{r^{2}}{2{\left(\frac{{\text{σ}}_{xy}}{2\sqrt{2\text{ln}2}}\right)}^{2}}\right)\left(1-\mathrm{re}\right)\text{exp}\left(-\alpha \left(\mathrm{th}-z\right)\right) \end{array}$$ (4)

where *th* is the thickness of the material model.

The simulation domain was defined as a rotationally symmetric 3-dimensional (3D) model (Fig. S3) with a radius of 500 μm and a thickness of 300 μm. The laser beam was centered on the top surface. Thermal radiation from the surface was neglected due to the low surface temperature. The initial temperature was set to 298.15 K, and natural convection boundary conditions were applied to the top surface. To accurately capture the light absorption and heat diffusion near the laser spot, a high-density mesh was used at the center of the top surface (Fig. S4).

For the aluminum-coated model, a 90-nm Al film was placed on the top surface (Fig. S5). A thermal contact interface was defined between the Al film and BAs substrate with an interfacial thermal conductance as another fitting parameter. Because of the thinness of the Al layer, an extremely fine mesh was applied within the film to resolve the light absorption and subsequent heat diffusion accurately (Fig. S6).

## Data Availability

The original data in this work are available from the corresponding authors upon reasonable requests.

## References

[1] Zhao DL, Qian X, Gu XK, Jajja SA, Yang RG. Measurement techniques for thermal conductivity and interfacial thermal conductance of bulk and thin film materials. J Electron Packag. 2016;138(4): Article 040802.

[2] Jiang PQ, Qian X, Yang RG. Tutorial: Time-domain thermoreflectance (TDTR) for thermal property characterization of bulk and thin film materials. J Appl Phys. 2018;124(16): Article 161103.

[3] Cahill DG, Fischer HE, Klitsner T, Swartz ET, Pohl RO. Thermal-conductivity of thin-films—Measurements and understanding. J Vac Sci Technol A Vac Surf Films. 1989;7(3):1259–1266.

[4] Yang J, Ziade E, Schmidt AJ. Uncertainty analysis of thermoreflectance measurements. Rev Sci Instrum. 2016;87(1): Article 014901. 26827342 10.1063/1.4939671

[5] Kang JS, Li M, Wu H, Nguyen H, Hu Y. Experimental observation of high thermal conductivity in boron arsenide. Science. 2018;361(6402):575–578. 29976798 10.1126/science.aat5522

[6] Li S, Zheng Q, Lv Y, Liu X, Wang X, Huang PY, Cahill DG, Lv B. High thermal conductivity in cubic boron arsenide crystals. Science. 2018;361(6402):579–581. 29976796 10.1126/science.aat8982

[7] Tian F, Song B, Chen X, Ravichandran NK, Lv Y, Chen K, Sullivan S, Kim J, Zhou Y, Liu T-H, et al. Unusual high thermal conductivity in boron arsenide bulk crystals. Science. 2018;361(6402):582–585. 29976797 10.1126/science.aat7932

[8] Qian X, Ding ZW, Shin JW, Schmidt AJ, Chen G. Accurate measurement of in-plane thermal conductivity of layered materials without metal film transducer using frequency domain thermoreflectance. Rev Sci Instrum. 2020;91(6): Article 064903. 32611038 10.1063/5.0003770

[9] Warkander S, Wu JQ. Transducerless time domain reflectance measurement of semiconductor thermal properties. J Appl Phys. 2022;131(2): Article 025101.

[10] Wang L, Cheaito R, Braun JL, Giri A, Hopkins PE. Thermal conductivity measurements of non-metals via combined time- and frequency-domain thermoreflectance without a metal film transducer. Rev Sci Instrum. 2016;87(9): Article 094902. 27782592 10.1063/1.4962711

[11] Shin J, Gamage GA, Ding ZW, Chen K, Tian F, Qian X, Zhou JW, Lee H, Zhou JS, Shi L, et al. High ambipolar mobility in cubic boron arsenide. Science. 2022;377(6604):437–440. 35862526 10.1126/science.abn4290

[12] Yue S, Tian F, Sui XY, Mohebinia M, Wu XX, Tong T, Wang ZM, Wu B, Zhang Q, Ren ZF, et al. High ambipolar mobility in cubic boron arsenide revealed by transient reflectivity microscopy. Science. 2022;377(6604):433–436. 35862517 10.1126/science.abn4727

[13] Tian ZY, Zhang QY, Xiao YW, Gamage GA, Tian F, Yue S, Hadjiev VG, Bao JM, Ren ZF, Liang E, et al. Ultraweak electron-phonon coupling strength in cubic boron arsenide unveiled by ultrafast dynamics. Phys Rev B. 2022;105(17): Article 174306.

[14] Bao JM, Pfeiffer LN, West KW, Merlin R. Ultrafast dynamic control of spin and charge density oscillations in a GaAs quantum well. Phys Rev Lett. 2004;92(23): Article 236601. 15245181 10.1103/PhysRevLett.92.236601

[15] Matatagui E, Thompson AG, Cardona M. Thermoreflectance in semiconductors. Phys Rev. 1968;176(3):950.

[16] Chelikowsky JR, Cohen ML. Nonlocal pseudopotential calculations for the electronic structure of eleven diamond and zinc-blende semiconductors. Phys Rev B. 1976;14(2):556–582.

[17] Chen X, Li CH, Tian F, Gamage GA, Sullivan S, Zhou JS, Broido D, Ren ZF, Shi L. Thermal expansion coefficient and lattice anharmonicity of cubic boron arsenide. Phys Rev Appl. 2019;11(6): Article 064070.

[18] Green MA, Keevers MJ. Optical-properties of intrinsic silicon at 300 k. Prog Photovoltaics. 1995;3(3):189–192.

[19] Aspnes DE, Studna AA. Dielectric functions and optical-parameters of Si, Ge, GaP, GaAs, GaSb, InP, InAs, and InSb from 1.5 to 6.0 eV. Phys Rev B. 1983;27(2):985–1009.

[20] Flubacher P, Leadbetter AJ, Morrison JA. The heat capacity of pure silicon and germanium and properties of their vibrational frequency spectra. Philos Mag. 1959;4(39):273–292.

[21] Victor AC. Heat capacity of diamond at high temperatures. J Chem Phys. 1962;36(7):1903.

[22] Vasil’ev VP, Gachon JC. Thermodynamic properties of InP. Inorg Mater. 2006;42(11):1171–1175.

[23] Yue S, Gamage GA, Mohebinia M, Mayerich D, Talari V, Deng Y, Tian F, Dai SY, Sun H, Hadjiev VG, et al. Photoluminescence mapping and time-domain thermo-photoluminescence for rapid imaging and measurement of thermal conductivity of boron arsenide. Mater Today Phys. 2020;13: Article 100194.

[24] Kang JS, Li M, Wu H, Nguyen HD, Hu YJ. Basic physical properties of cubic boron arsenide. Appl Phys Lett. 2019;115(12): Article 122103.

[25] Jaramillo-Fernandez J, Chavez-Angel E, Sanatinia R, Kataria H, Anand S, Lourdudoss S, Sotomayor-Torres CM. Thermal conductivity of epitaxially grown InP: Experiment and simulation. CrystEngComm. 2017;19(14):1879–1887.

[26] Dames C. Ultrahigh thermal conductivity confirmed in boron arsenide. Science. 2018;361(6402):549–550. 30093587 10.1126/science.aau4793

[27] Di Cicco A, Polzoni G, Gunnella R, Trapananti A, Minicucci M, Rezvani SJ, Catone D, Di Mario L, Pelli Cresi JS, Turchini S, et al. Broadband optical ultrafast reflectivity of Si, Ge and GaAs. Sci Rep. 2020;10(1):17363. 33060665 10.1038/s41598-020-74068-y PMC7567120

[28] Shimofuri M, Murakami T, Miyake S, Banerjee A, Hirotani J, Tsuchiya T. Numerical calculation of thermoreflectance coefficient of c-Si for wavelengths of 200-800 nm and temperatures of 300-500 K. Jpn J Appl Phys. 2023;62(11): Article 112006.

[29] Zhong H, Pan FJ, Yue SA, Qin CZ, Hadjiev V, Tian FA, Liu XF, Lin F, Wang ZM, Bao JM. Idealizing Tauc plot for accurate bandgap determination of semiconductor with ultraviolet-visible spectroscopy: A case study for cubic boron arsenide. J Phys Chem Lett. 2023;14(29):6702–6708. 37467492 10.1021/acs.jpclett.3c01416

[30] Kiliani D, Micard G, Steuer B, Raabe B, Herguth A, Hahn G. Minority charge carrier lifetime mapping of crystalline silicon wafers by time-resolved photoluminescence imaging. J Appl Phys. 2011;110(5): Article 054508.

[31] Dai SY, Xing XX, Hadjiev VG, Qin ZJ, Tong T, Yang G, Wang C, Hou LJ, Deng LZ, Wang ZM, et al. Theory and experiments of pressure-tunable broadband light emission from self-trapped excitons in metal halide crystals. Mater Today Phys. 2023;30: Article 100926.

[32] Assali S, Zardo I, Plissard S, Kriegner D, Verheijen MA, Bauer G, Meijerink A, Belabbes A, Bechstedt F, Haverkort JEM, et al. Direct band gap wurtzite gallium phosphide nanowires. Nano Lett. 2013;13(4):1559–1563. 23464761 10.1021/nl304723c PMC3624814

[33] Chen X, Li CH, Xu YM, Dolocan A, Seward G, Van Roekeghem A, Tian F, Xing J, Guo SC, Ni N, et al. Effects of impurities on the thermal and electrical transport properties of cubic boron arsenide. Chem Mat. 2021;33(17):6974–6982.

[34] Fava M, Protik NH, Li CH, Ravichandran NK, Carrete J, van Roekeghem A, Madsen GKH, Mingo N, Broido D. How dopants limit the ultrahigh thermal conductivity of boron arsenide: A first principles study. npj Comput Mater. 2021;7(1):54.

[35] Hou SR, Pan FJ, Shi XP, Angeles F, Gamage GA, Sun HR, Niyikiza BA, Nataj ZE, Kargar F, Balandin AA, et al. Strong temperature dependence of thermal conductivity in high-purity cubic boron arsenide. Phys Rev B. 2025;111(23): Article 235203.

[36] Niyikiza AB, Xiang ZY, Zhang FH, Pan FJ, Li C, Broido D, Peng Y, Liao BL, Ren ZF. Thermal conductivity of boron arsenide above 2100 watts per meter per kelvin at room temperature. arXiv. 2025. 10.48550/arXiv.2505.14434

