# Supplementary material for: Thermal Conductivity above 2,000 W/m·K in Boron Arsenide by Nanosecond Transducer-Less Time-Domain Thermoreflectance
Source: Research (Wash D C). 2025 Oct 27;8:0971. doi: 10.34133/research.0971 (PMC12554925; doi:10.34133/research.0971)
Supplement: Supplementary 1 — Figs. S1 to S13 Table S1 [file research.0971.f1.docx]

**Thermal Conductivity above 2000 W/m·K in Boron Arsenide by Nanosecond Transducer-less Time-Domain Thermoreflectance**

Hong Zhong^1†^, Ying Peng^2†^, Feng Lin^1†^, Ange Benise Niyikiza^2^, Fengjiao Pan^2^, Chengzhen Qin^3^, Jinghong Chen^1^, Viktor G. Hadjiev^4^, Liangzi Deng^1^, Zhifeng Ren^2*^, Jiming Bao^1,2,3*^

^1^Department of Electrical & Computer Engineering and Texas Center for Superconductivity at the University of Houston (TcSUH), University of Houston, Houston, Texas 77204, USA.

^2^Department of Physics and Texas Center for Superconductivity at the University of Houston (TcSUH), University of Houston, Houston, Texas 77204, USA.

^3^Materials Science and Engineering, University of Houston, Houston, Texas 77204, USA.

^4^Department of Mechanical Engineering and Texas Center for Superconductivity at the University of Houston (TcSUH), University of Houston, Houston, Texas 77204, USA.

^†^These authors contributed to the work equally

^*^Corresponding authors. Email: zren@uh.edu, jbao@uh.edu


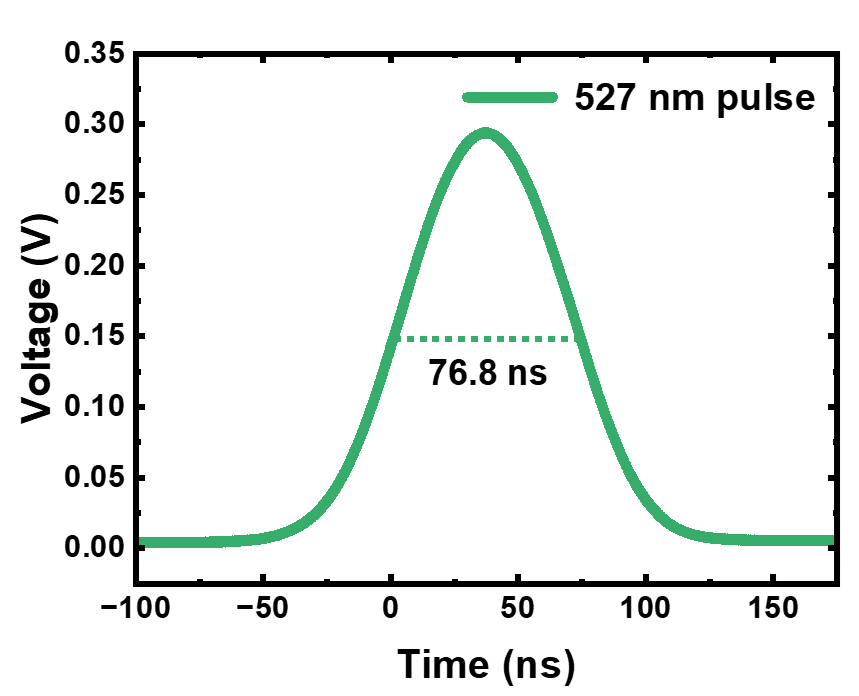


**Fig. S1.** Single pulse profile of the 527 nm pulsed laser. The full width at half maximum (FWHM) is measured to be 76.8 ns.


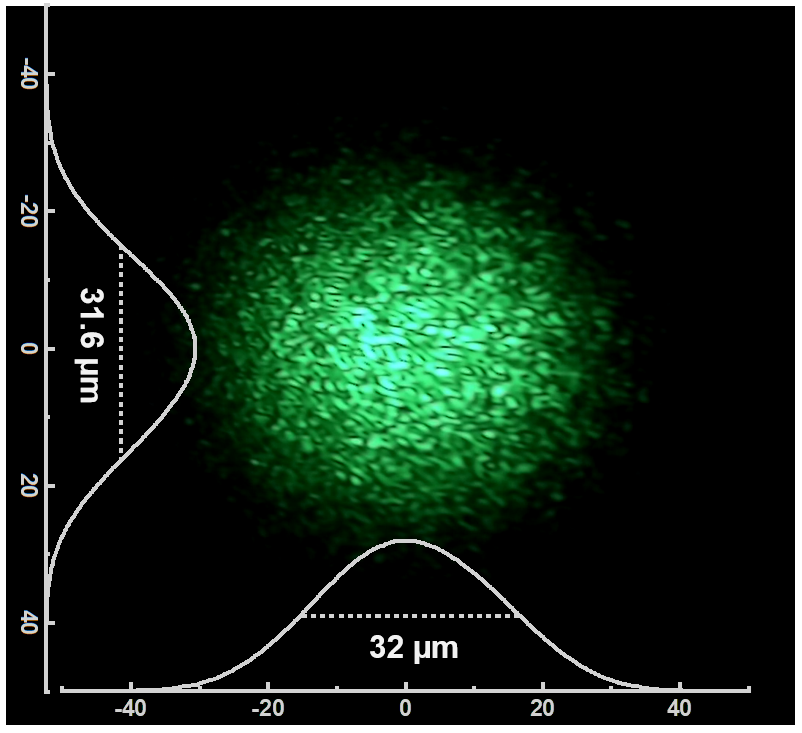


**Fig. S2.** Scattered spot image of 527 nm pulsed laser. Image captured by laser irradiating sandpaper with a particle size of 0.1 µm. Intensity profiles along the horizontal and vertical directions were extracted and fitted with Gaussian functions, yielding spot sizes of 32 µm and 31.6 µm, respectively. The final spot size was taken as the average value of 31.8 µm.

**Table S1.** Input simulation parameters

| **Parameters**    **Materials** | ***ρ***  ***(kg/m^3^)*** | ***C_p_***  ***(J/kg·K)*** | ***Sigma.xy***  ***σ_xy_***  ***(μm)*** | ***Sigma.t***  ***σ_t_***  ***(ns)*** | ***α***  ***(1/m)*** |
| --- | --- | --- | --- | --- | --- |
| **BAs** | 5220 | 408 **^1^** | 31.8 | 76.8 | 50,000 **^7^** |
| **Si** | 2329 | 700 **^2^** | 31.8 | 76.8 | 880,000 **^5,6^** |
| **Diamond** | 3500 | 519 **^3^** | 31.8 | 80 | NA |
| **InP** | 4810 | 310 **^4^** | 31.8 | 80 | 11,300,000 **^5^** |
| **Ge** | 5327 | 322 **^2^** | 31.8 | 80 | 45,000,000 **^5^** |

***ρ***  Density of material

***C_p_*** Specific heat capacity of material

***Sigma.xy (σ_xy_*)** Diameter of pump laser spot

***Sigma.t (σ_t_***) Pulse width of pump laser

***α*** Absorption coefficient at 527 nm

***d*** Diameter of prober laser spot

[1] X. Chen, C. H. Li, F. Tian, G. A. Gamage, S. Sullivan, J. S. Zhou, D. Broido, Z. F. Ren, L. Shi., Thermal Expansion Coefficient and Lattice Anharmonicity of Cubic Boron Arsenide, Phys. Rev. Appl. 11, 064070 (2019).

[2] P. Flubacher et al. The heat capacity of pure silicon and germanium and properties of their vibrational frequency spectra. Philos. Mag.‑J. Theor. Exp. Appl. Phys. 4, 273 (1959).

[3] A. C. Victor, Heat Capacity of Diamond at High Temperatures, J. Chem. Phys. 36, 1903 (1962)

[4] V. P. Vasil’ev et al., Thermodynamic properties of InP, Inorg. Mater. 42, 1171 (2006)

[5] D. E. Aspnes, Dielectric functions and optical parameters of Si, Ge, GaP, GaAs, GaSb, InP, InAs, and InSb from 1.5 to 6.0 eV, Phys. Rev. B, 27, 985 (1983)

[6] M. A. Green, M. J. Keevers, Optical-properties of intrinsic silicon at 300 k, Prog. Photovoltaics 3, 189 (1995).

[7] S. Yue et al., Photoluminescence mapping and time-domain thermo-photoluminescence for rapid imaging and measurement of thermal conductivity of boron arsenide. Materials Today Physics 13, 100194 (2020).


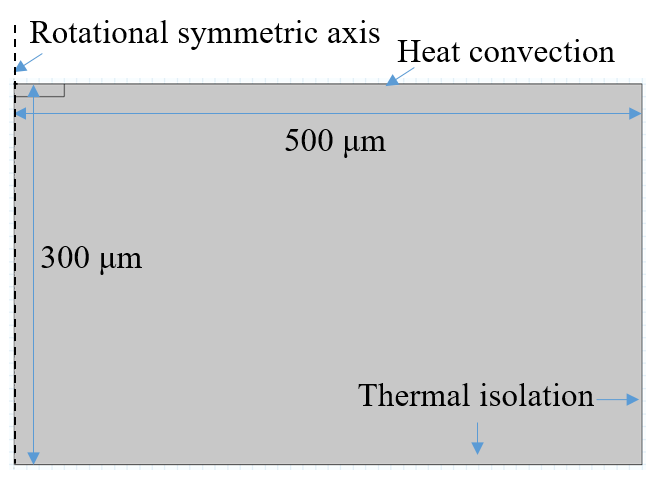


**Fig. S3** The rotationally symmetric simulation model of BAs.


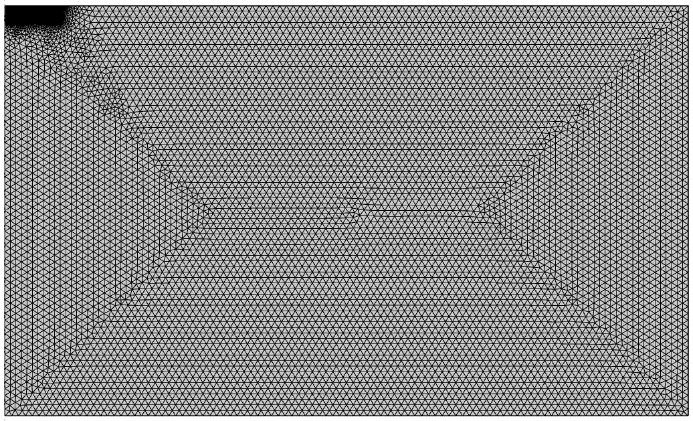


**Fig. S4** The mesh of simulation model.


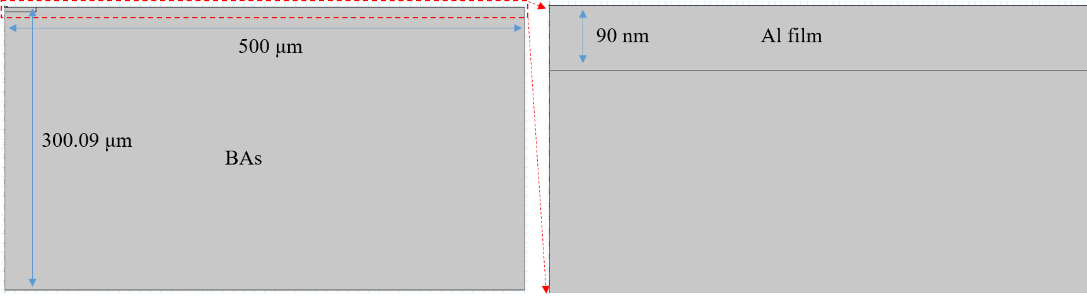


**Fig. S5** Simulation model of Al-film coated BAs.


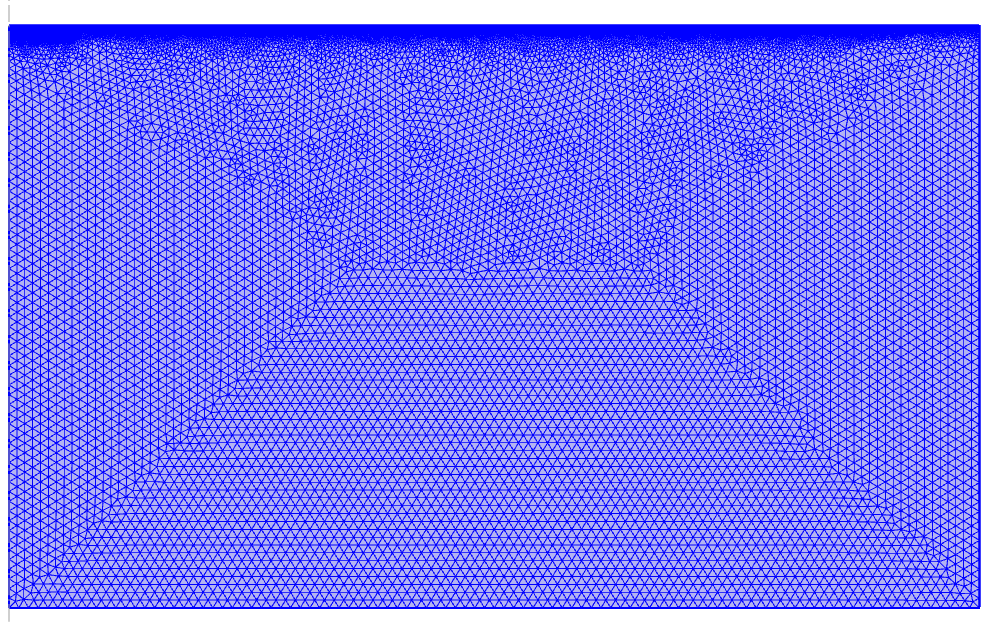


**Fig. S6.** The mesh of Al-film coated model.

**Fig. S7.** Temperature dependent reflectance of Si at 450 nm.

**Fig. S8.** Temperature dependent reflectance of BAs at 450 nm.

**Fig. S9.** Sensitivity of Si tl-TDTR ΔR/R to pump spot size (diameter σ_xy_ = 31.8 µm), pump pulse width (σ_t_ = 76.8 ns) and pump optical absorption coefficient at 527 nm (α = 8.8 × 10^5^ m⁻¹). The thermal conductivity of Si is set to 150 W/m·K. Other parameters of Si can be found from the Table S1.

**Fig. S10**. Uncertainties in thermal conductivity of silicon due to uncertainties in pump pulse width (76.8 ns ±2%), pump pulse spot size (31.8 µm ±5%) and pump absorption coefficient (8.8 × 10^5^ m⁻¹ ±22%).

Fig. S11. Uncertainties in thermal conductivity of BAs due to uncertainties in pump pulse width (76.8 ns ±2%) and pump pulse spot size (31.8 µm ±5%).

Fig. S12. Temperature-dependent thermal conductivity of the spot P1 from the sample in Fig. 2, along with the data extracted from the paper Ref. 35, Hou et al. Phys. Rev. B 111, 235203 (2025). The temperature dependent heat capacity is taken from Ref. 24, J. S. Kang, M. Li, H. Wu, H. Nguyen, and Y. Hu, Basic physical properties of cubic boron arsenide, Appl. Phys. Lett. 115, 122103 (2019).


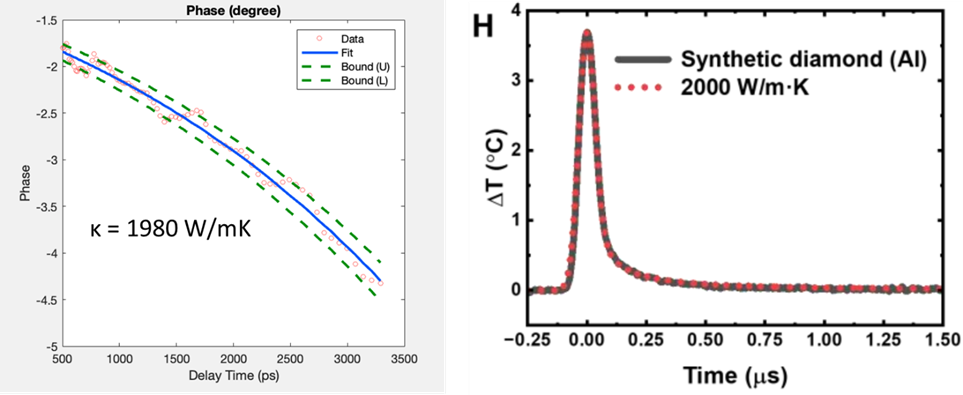


Fig. S13. Validation of the thermal conductivity of a synthetic diamond by (a) the traditional femtosecond TDTR (UCSB) and (b) our nanosecond tl-TDTR. Al film thickness: 74 nm. Interfacial resistance: 77.2 MW/m2K for (a) and 75 MW/m2K for (b).
